# Adaptive Imaging Cytometry to Estimate Parameters of Gene Networks Models in Systems and Synthetic Biology

**DOI:** 10.1371/journal.pone.0107087

**Published:** 2014-09-11

**Authors:** David A. Ball, Matthew W. Lux, Neil R. Adames, Jean Peccoud

**Affiliations:** Virginia Bioinformatics Institute, Virginia Tech, Blacksburg, VA, United States of America; Tata Institute of Fundamental Research, India

## Abstract

The use of microfluidics in live cell imaging allows the acquisition of dense time-series from individual cells that can be perturbed through computer-controlled changes of growth medium. Systems and synthetic biologists frequently perform gene expression studies that require changes in growth conditions to characterize the stability of switches, the transfer function of a genetic device, or the oscillations of gene networks. It is rarely possible to know *a priori* at what times the various changes should be made, and the success of the experiment is unknown until all of the image processing is completed well after the completion of the experiment. This results in wasted time and resources, due to the need to repeat the experiment to fine-tune the imaging parameters. To overcome this limitation, we have developed an adaptive imaging platform called GenoSIGHT that processes images as they are recorded, and uses the resulting data to make real-time adjustments to experimental conditions. We have validated this closed-loop control of the experiment using galactose-inducible expression of the yellow fluorescent protein Venus in *Saccharomyces cerevisiae*. We show that adaptive imaging improves the reproducibility of gene expression data resulting in more accurate estimates of gene network parameters while increasing productivity ten-fold.

## Introduction

Quantitative time-lapse microscopy, or imaging cytometry, has become a tool of choice to characterize the dynamics of gene networks in individual cells [1]–[3], because it allows the study of cell-to-cell heterogeneity (noise) of the network rather than just the average behavior [4]. Systems biologists have been using this technique to collect data used to better understand specific aspects of natural regulatory networks. For instance, imaging of live yeast cells was instrumental to observe and understand the impact of molecular noise on the timing of cell division [5], the coherence [6], [7] and irreversibility [8] of the start transition. It was also used to validate a mathematical model of the cell cycle regulatory network [9] and to measure the periodic expression of proteins involved in the control of cell division [10]. Still in yeast, time-lapse microscopy has recently been used to uncover short-term epigenetic effects affecting transcription [11] and support the development of a model explaining how yeast cells modify their environment to increase mating efficiency [12].

Imaging cytometry has also been crucial for studying network dynamics in organisms other than yeast. For example, a recent review describes the pervasiveness of pulsatile dynamics across many species that has been revealed by time-lapse microscopy [13]. In mammalian studies, imaging cytometry has been used to study diverse processes including the dynamics of cell signaling in the Ras/Erk system [14], the role of feedback loops in differentiation [15], and chemotaxis [16]. Synthetic biologists have also turned to imaging cytometry to study engineered genetic clocks [17], [18], and improved coupling between various genetic circuits based on overloaded protein degradation machinery [19].

The incorporation of microfluidic systems further enhance live-cell imaging by allowing the biological system to be actively perturbed with the addition or removal of chemical signals, while the cells remain under observation [20]–[22]. Computer-controlled microfluidics have thus far been used to change the cell environment in a periodic fashion for the study of oscillators [17], [18], but they also hold the promise of allowing precise control over the time when chemicals are added to perform such experiments as gene induction. Such changes of environments are generally programmed at times specified prior to starting the image acquisition process.

This approach to performing experiments is problematic because the operator initially does not know the specific dynamics of the network that is to be studied. It is therefore common to have to repeat the experiment multiple times until the time resolution of the image acquisition and the changes of medium are tuned to match the dynamics of the system under observation. This trial-and-error approach is wasteful of time and resources. Furthermore, in comparison to flow cytometry, imaging cytometry incurs limited throughput (i.e. the number of cells that can be observed with a given time resolution) because of hardware latencies, such as focusing, and sample exposure times.

One option to overcome these problems is to develop an adaptive imaging cytometry platform that analyzes images as they are collected, and uses the processed information to automatically adjust the parameters of the experiment. Jelena Kovacevic first proposed the idea of optimizing image acquisition processes by adapting imaging conditions in real-time in order to maximize various figures of merit [23]–[26] but this line of research has remained theoretical so far due to the lack of instruments capable of implementing such algorithms. Here, we present GenoSIGHT, the first imaging system relying on a closed-loop control algorithm to adapt the collection of a series of time-lapse images to optimize the measurement of gene expression data in individual cells. This is achieved by first optimizing the selection of areas on the slide to be imaged, also known as Fields of View (FOV), that will be used to collect time-lapse series of images. After this selection, the closed-loop control is used to change the growth medium and the time resolution of the image acquisition in response to the dynamics of gene expression dynamics observed in the cell population.

## Materials and Methods

### Yeast Strains and Media

A yeast strain (K699 *MAT*a *ade2-1 trp1-1 can 1-100 leu2::LEU2-GAL1pr-VYFP his3-11,15 ura3*) expressing genomically-inserted Venus yellow fluorescent protein (vYFP) under the control of the inducible *GAL1* promoter was generously provided by the O’Shea lab (Harvard University, USA) [27]. All imaging experiments were conducted in Synthetic Complete (SC) medium with 2% raffinose, and then SC+2% galactose was used to induce expression. The translation inhibitor cycloheximide (final concentration of 20 µg/ml) was used to block protein production to quantify maturation of the vYFP proteins.

### Hardware

All images were collected on an Axio Observer Z1 microscope (Carl Zeiss Microscopy, LLC), which is equipped with a halogen lamp for bright-field mode, and a 120 W Metal Halide lamp (Lumen Dynamics Group, Inc., model: X-Cite 120PC Q) for fluorescence excitation. The microscope is fully automated, including a linear-encoded x-y translation stage (Ludl Electronics Products, Ltd., model: 96S108-LE), filter wheel, shutters, and is equipped with a CoolSNAP HQ camera (Photometrics, 6.45 µm pixels, 1392×1040 resolution). A 63× glycerol-immersion, phase-contrast objective (N.A. 1.3) was used to collect both phase contrast and fluorescence images. A GFP filter-set (Chroma Technology Corp., set 49002) with the excitation band centered at 470 nm (full-width of 40 nm) and emission band centered at 525 nm (full-bandwidth of 50 nm) was used to image Venus-expressing cells with an exposure time of 75 ms. The computer-controllable ONIX microfluidic system (EMD Millipore, model: EV-262) was used to trap cells, provide them with a continuous flow of fresh media, and to change media during the experiment.

All hardware control and image-processing is performed on a PC running Windows 7 with 4 GB of RAM, and a dual core, 32-bit Intel i5 processor.

### Software

The GenoSIGHT software was developed in MATLAB and is distributed using the Apache 2.0 license and is available from SoureForge (http://sourceforge.net/projects/genosight). All communication with the hardware was handled through the µManager API (version 1.4.14), which is an open-source microscopy control application [28]. The Java-based software allows direct control of all components, and after some initial setup, can be called directly from MATLAB. Although, GenoSIGHT has only been tested with the hardware described above, it uses a hardware configuration file created by µManager, which supports a multitude of components, and so GenoSIGHT should be compatible with most hardware setups.

The autofocusing in GenoSIGHT is performed in software, and is based on image contrast [29], [30]. The system first collects an image at 5 positions along the z-axis: 2 above, 2 below, and 1 at the current z-position, separated by 2 µm. A contrast metric, C, is calculated for each plane based on the autocorrelation:
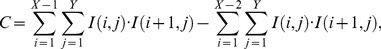
(1)where *X* and *Y* are the dimensions in pixels of the image, *I*. The system moves to the plane that maximizes C, and then repeats the process by collecting 5 more images along the z-axis separated by a finer step-size of 0.3 µm. The optimal focus is then defined as the plane from this second set that again maximizes the contrast.

Phase-contrast images are segmented using custom software that relies on the MATLAB Image Processing toolbox. First, the function ‘imfill’ is used to flood fill local minimum not connected to the image border, which fills in the center of the groups of cells. As each group of cells will have slightly different levels to which the flood fill will rise, we then search the image histogram for intensities greater than the calculated background, taken from the border pixels, and that occur with a frequency greater than the minimum cell area, generally set to 200 pixels. To keep only large groups of connected pixels, erosion (built-in function ‘imerode’) is performed, removing the outermost pixels of a region and eliminating small groups of pixels (small bubbles or debris).

The next step is to separate these groups into individual cells. This is done with another call to ‘imerode’ to cut the small necks that appear between touching cells. Once the cells are cut, the remaining connected regions are labeled with a call to the built-in function ‘bwlabel’, which identifies the individual cells and assigns each with a unique label. To finish, the cells are returned to their original sizes with a dilation (built-in function ‘imdilate’), which adds pixels around the edges of each cell.

After an image is collected, the pixels making up each cell body are mapped to the previous frame by calculating the overlap (defined here as the ratio of the intersection of cell-body pixels to their union) of the current cell with the cells in the previous frame. The processing time required to complete the segmentation depends on the number of cells in the image, but is typically on the order of one second, making it feasible to perform in real time.

We have compared the performance of the above algorithm to CellTracer [31], as it is also implemented in MATLAB, and was easily integrated into GenoSIGHT. Figure S1 shows the speed and performance of CellTracer compared to GenoSIGHT’s native image processing. Although CellTracer is better at identifying cells in crowded images, the time for cell identification increases linearly with the number of cells in the image, meaning that the time-resolution for an adaptive experiment would be further degraded.

### Data Analysis

Prior to fitting the maturation and transcriptional memory data, the raw cell trajectories are filtered to remove any cell that was not present for at least 50 time points (∼250 min.), which is the value reported in Table 1. The mean fluorescence trajectory was calculated by averaging the fluorescence of all remaining cells at each time-point. For curve-fitting of the maturation data, each average fluorescence curve was normalized between 0 and 1, by first subtracting the minimum value that occurs in the curve, and then dividing by the maximum value.

**Table 1:** Summary of *GAL1-Venus* induction experiments.

	Adaptive				Conventional Fixed Times			
	Cell Growth (min)	Gal Induction (min)	Cells	*Maturation Half-time (min)*	Cell Growth (min)	Gal Induction (min)	Cells	*Maturation Half-time (min)*
Exp. 1	61.6	42.4	333	*15.1*	63.7	29.7	342	*9.7*
Exp. 2	75.7	42.4	705	*17.2*	62.8	31.4	466	*17.4*
Exp. 3	91.7	36.6	167	*13.3*	60.8	30.3	470	*17.2*
Mean (SEM*)	76.3 (8.69)	40.5 (1.93)	402	*15.2 (1.11)*	62.9 (0.76)	29.7 (0.82)	421	*14.8 (2.51)*
CV**	0.20	0.08	0.69	*0.13*	0.02	0.06	0.14	*0.29*

*SEM = standard error of the mean (N = 3).

**CV = coefficient of variation.

## Results

### Automated selection of Fields-of-View

When attempting to track many cells at the fastest time resolution possible, it is crucial to select FOVs that contain an optimal number of cells for time-lapse imaging. Obviously, FOVs that contain no cells should be ignored. On the other hand, if a FOV has too many cells, the FOV will become overcrowded as cells grow and divide, causing difficulty in detecting individual cells.

We have automated the process of FOV selection by incorporating the image processing into the instrument control. First, the user specifies the number of desired FOVs, *N*, and specifies a plane in three dimensions by marking x, y, and z coordinates of the top-left, top-right, and bottom-right corners of the area to search. While scanning through x and y directions, the plane equation is used to calculate the optimal z position. This is done to avoid autofocusing after each move, which takes roughly four seconds at each FOV, and quickly becomes impractical when scanning thousands of FOVs.

**Figure 1:**
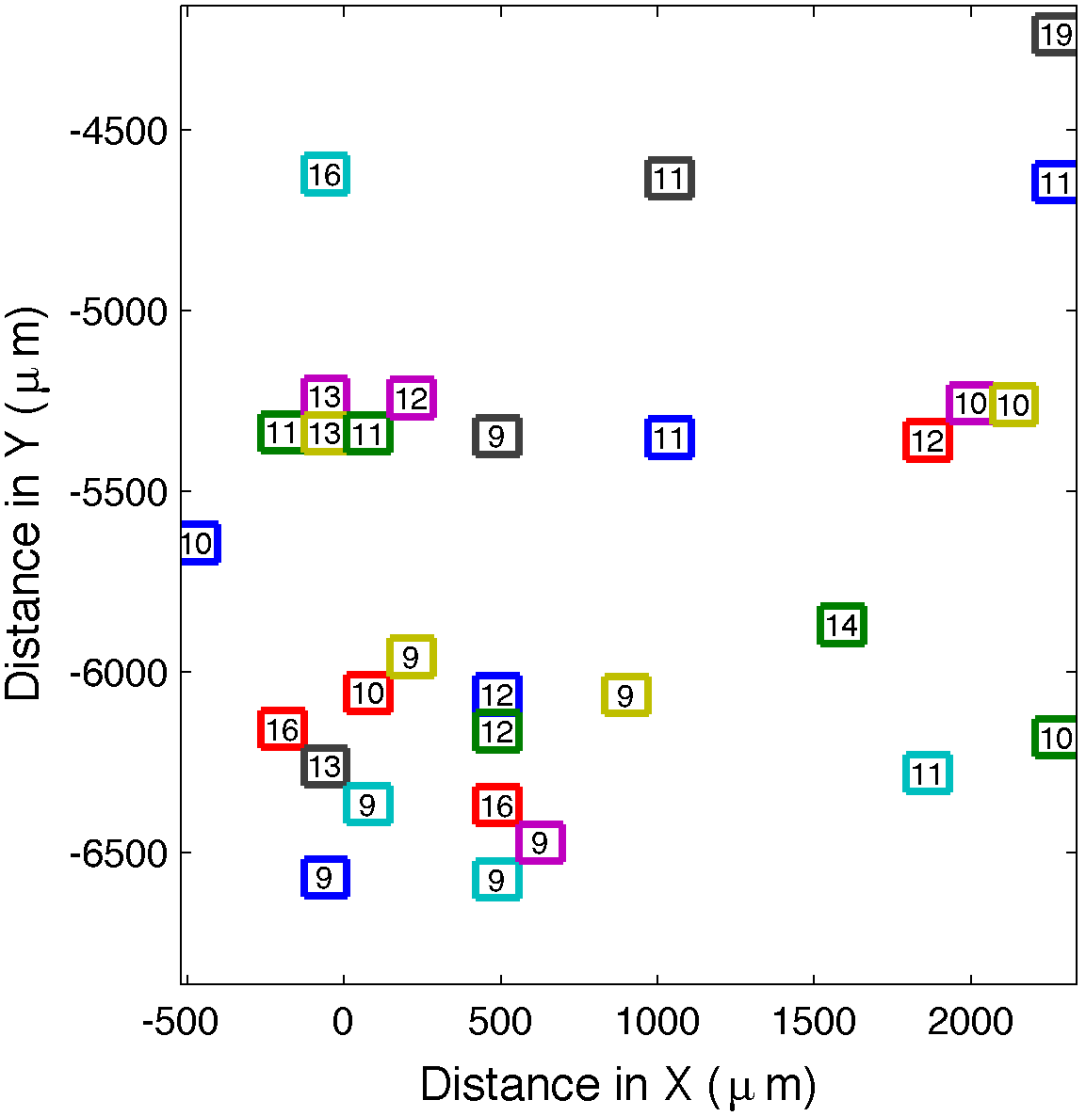
Automatically identified Fields-of-View. The optimal FOVs from a 3×3 mm micro-fluidic chamber automatically found by GenoSIGHT with a user-supplied maximum of 20 cells in any one FOV. The number of cells in each FOV is indicated inside of each colored rectangle.

Phase contrast images are taken and processed to count the number of cells at each position within the user-defined area as the sample is moved in steps equal to the size of the camera sensor in object space (physical size/magnification). Currently, the pixel size, 6.45 µm (from the camera specifications) is hard-coded into GenoSIGHT, but the software captures the number of pixels in each direction, the pixel binning, and the magnification from the Graphical User Interface. The coordinates of any FOV that contains at least one cell, but less than a user-defined threshold (typically 20 cells) is saved to memory along with the number of cells in that FOV. After the scanning is completed, the FOVs are sorted in order of decreasing number of cells, and only the first *N* FOVs are kept to maximize the number of tracked cells. These remaining positions are then reordered to minimize the distance that the translation stage has to move. Figure 1 shows 30 FOVs automatically selected by GenoSIGHT from a scan of the entire 3 mm×3 mm trapping area of a micro-fluidic device. This figure shows that the FOVs selected by GenoSIGHT are scattered throughout the entire region specified by the operator rather than limited to one portion of the chamber as would be typical from manually selected FOVs. The number of cells in each FOV is also narrowly distributed.

The time required for automated scanning is dependent on the size of the scan area, and for the area depicted in Figure 1, which contains 588 FOVs at 63x, the scan took ∼20 minutes.

### Determining the maximum time resolution

Once the FOVs have been identified, it is possible to determine the maximum time resolution applicable for these FOVs. In order to maximize the amount of data collected in an imaging experiment, it is desirable to minimize the amount of time that the microscope is idle. Because there is an inherent trade-off between number of FOVs and the frequency at which they can be imaged, the only way to maximize the throughput (cells×time-points) is to fully characterize the hardware and software latencies of the imaging process.

**Figure 2:**
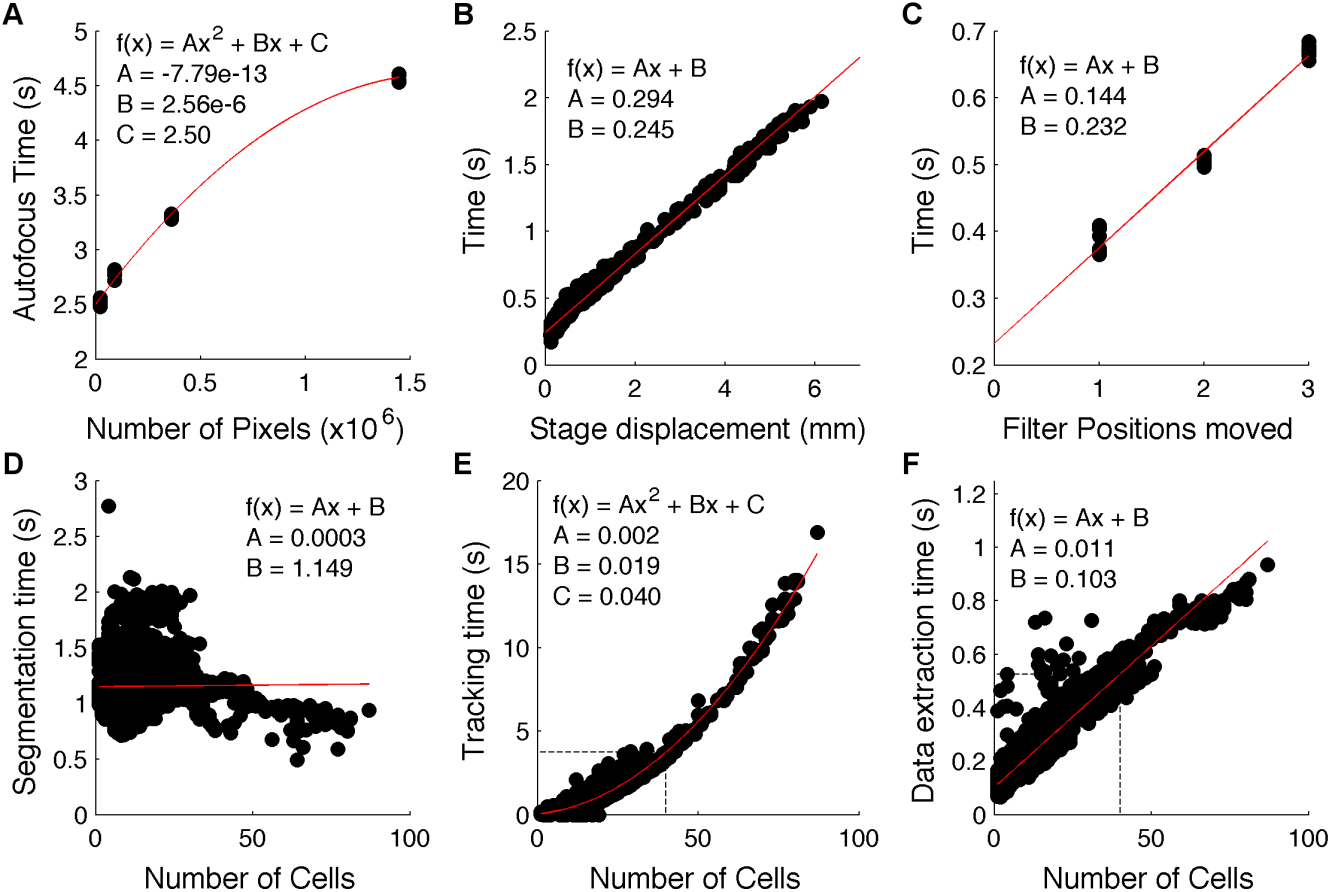
Hardware & software latencies. The time taken to (A) autofocus on a field-of-view with a CCD of various areas, (B) move the sample translation stage a given distance, (C) change from 1 position in the filter cube to another, (D) identify all of the cells in an image, (E) track in time all of the cells in an FOV from the previous time-point, and (F) extract all size and fluorescence data from an image. In all plots, experimental measurements are shown as black dots, and red lines show the best-fit line to the data, with the polynomial coefficients inset. The dashed, black lines in (E) and (F) indicate the times that are used for *t_map_*, and *t_ext_*, respectively, in the calculation of 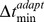.

The latencies are inherently dependent on the specific components used in the hardware setup, and we have therefore used the Profiler benchmarking tool in MATLAB to empirically measure the time that is required for each step in the image acquisition process for GenoSIGHT. The time-consuming steps include the time to autofocus (*t_AF_*, Figure 2A), the time needed for the sample stage to travel a specified distance *x* (*t_mot_* (*x*), Figure 2B), and the time needed to change from one filter position to another that is *k* positions away (*t_filt_* (*k*), Figure 2C). For an experiment with *N* FOVs and *P* channels (which could include multiple fluorescence images as well as the phase contrast images), the exposure times (*t_exp_*) along with the above values determine the minimum time resolution,
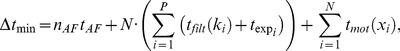
(2)where 0≤*n_AF_*≤*N* is the number of FOVs at which to autofocus. To estimate each contribution, each step was run separately many times with various parameters and recording the time needed to accomplish the procedure. For example, the autofocus time, tAF, was measured 5 times at each of the cameras resolution settings (pixels binned in groups of 1×1, 2×2, 4×4 and 8×8). Based on least-squares curve fitting of each contribution using the lowest-order polynomial function that could explain the behavior (R^2^>0.9, red lines in Figure 2), Equation 2 can be solved at run time. More specifically, once the user has defined the filters to be used and exposure times for each channel, and specified the FOVs to revisit, the two summations can be calculated. The time required for autofocus (see Materials and Methods for autofocusing details), *t_AF_*, can be calculated based on number of pixels in the image and the exposure time used to collect the phase contrast image. The image exposure time simply introduces an offset into the curve shown in Figure 2A, and so can be added to the value that is calculated from the indicated quadratic equation. The time resolution can be greatly reduced by autofocusing on a single FOV (*n_AF_* = 1), and propagating any displacement to the other FOVs.

For adaptive experiments, images are analyzed as they are collected, and the time needed to identify cells (*t_seg_*, Figure 2D), track cells (*t_map_*, Figure 2E), and extract data (*t_ext_*, Figure 2F) increases the minimum allowable resolution to
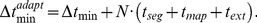
(3)


The segmentation is largely independent of cell number (*n*) due to the inherent use of parallelization by MATLAB’s image processing toolbox, and we therefore set this to a constant value *t_seg_* = 1.15 s, which is the average value from Figure 2D. The data extraction time increases linearly with *n*, and mapping cells from the current time-point to previous time-points increases as *n^2^*. Because the time-resolution now further depends on the number of cells in each FOV, and not just the number of FOVs, the delay between time-points will increase over time as the number of cells increases. For the calculation of *t_min_*, we therefore set *t_map_* = 3.74 s, and *t_ext_* = 0.53 s (dotted lines in Figures 2E & F, respectively), both of which are adequate to process images with 40 cells in them. This ensures that the cells can be observed for at least one doubling interval with the desired resolution, when FOVs with a maximum of 20 cells are initially selected.

For a typical 2-channel (phase contrast with 10 ms exposure, and 1 fluorescence image with 75 ms exposure) experiment using *S. cerevisiae* with 2×2 binning of the CCD pixels, and autofocusing on every FOV on our system we find 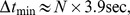 and 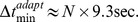 For the smaller *E. coli*, which requires the use of the full CCD without binning, and therefore more processing time, the software adds 40 seconds per FOV (data not shown). From Figure 2, it is clear that *t_map_* is the most significant contributor to 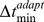
and therefore optimization of this step will offer the best opportunity as we work to improve the time resolution of adaptive imaging.

### Adaptive control algorithm

**Figure 3:**
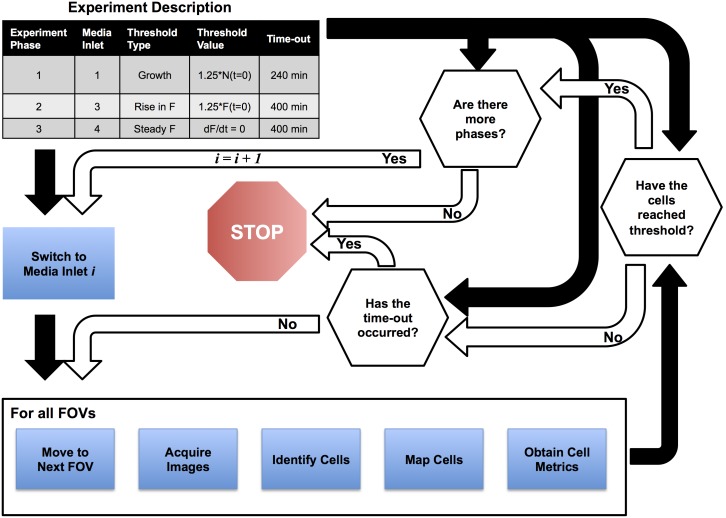
Adaptive control architecture. Prior to acquiring images, the user specifies various phases of the experiment that use different media inlets in the microfluidic system. Each phase has a criterion that determines when the system can proceed to the following phase, and a time-out that will end the experiment if encountered. After collecting and analyzing all FOVs for the current time-point, the system compares the individual cell data to the criterion. If the criterion is achieved, GenoSIGHT continues to the next phase, or ends the acquisition if the current phase is the last. If the criterion is not reached, and the time-out has not happened, the imaging continues.

For adaptive imaging, images are collected, and then immediately processed to identify and track cells and their fluorescence levels. Figure 3 depicts how GenoSIGHT uses this information to control in real time the cells’ environment. The user can define an experiment protocol, which is divided into different phases as shown in the upper left corner of Figure 3. In each phase, the user specifies a criterion to stop the current phase and move to the next, or stop the experiment if it is the last phase. The criteria are based on either changes in the number of cells present or fluorescence intensity, and each criterion is specified as a MATLAB function, making it possible to easily add new criteria as needed. For most of the criteria, the data from each time point is then compared to the user specified time point, which can be either the first time point of the experiment, the first time point of the current phase, or the previous time point. If the fractional change is greater than a user-specified threshold for the majority of the cells, then the phase will end. Each phase also has an adjustable time-out parameter, and if the duration of the phase reaches the specified time-out, then the experiment is ended. When the experiment ends, whether successful or not, the system notifies the experimenter of the outcome by email or text message.

### Application 1: estimation of fluorescent protein maturation rate

We first performed an experiment to characterize the maturation rate of the fluorescent protein Venus. The simplicity of the protocol allowed for a comparison of the adaptively collected time-course with that of non-adaptive imaging (i.e. media and chemicals were introduced at times specified by operator before the experiment started). The purpose of this experiment was primarily to ensure that changing the acquisition based on cell behavior did not introduce any artifacts in the data.

The experiment relies on a yeast strain having the Venus gene under the control of the *GAL1* promoter. The experiment was broken down into three phases. First, the number of cells was monitored in synthetic complete (SC) medium with 2% raffinose until the cell count increased by 25% to ensure that cells were growing exponentially. Then the media was switched to SC with 2% galactose and the experiment continued until 25% of the cells’ average fluorescence had increased by greater than 25% of background autofluorescence levels. Finally, the media was switched to SC with 2% galactose and cycloheximide to inhibit protein production, and the experiment continued until the fluorescence of 60% of the cells had become steady, which was determined when the best fit line of the last 5 data points had a slope of 0.

Three experiments were conducted in adaptive mode as described above, and three in non-adaptive mode. For non-adaptive imaging experiments the cell culture was grown overnight in non-inducing raffinose media. After loading the cells into the microfluidic device, the cells were exposed to raffinose media for 60 minutes to allow them to acclimate to their new environment, after which galactose media was introduced to induce expression of Venus. Then, as described in Gordon, et al [32], after 30 minutes of induction, the translation inhibitor cycloheximide was added. The cells were monitored for an additional 4 hours after the addition of cycloheximide, as maturation rate measurements of different fluorescent proteins range from several minutes to several hours [32]–[38]. Each experiment contained 30 FOVs, and the time resolution was calculated as described above. The timing varied slightly from experiment to experiment due to the differing distances traveled while visiting each FOV, but was approximately 5 min/frame for each. Although the non-adaptive experiments could have been performed with a finer time resolution, as the image processing was not used to control the experiment, the timing was kept consistent with the adaptive experiments to avoid differences in photobleaching and phototoxicity.

**Figure 4:**
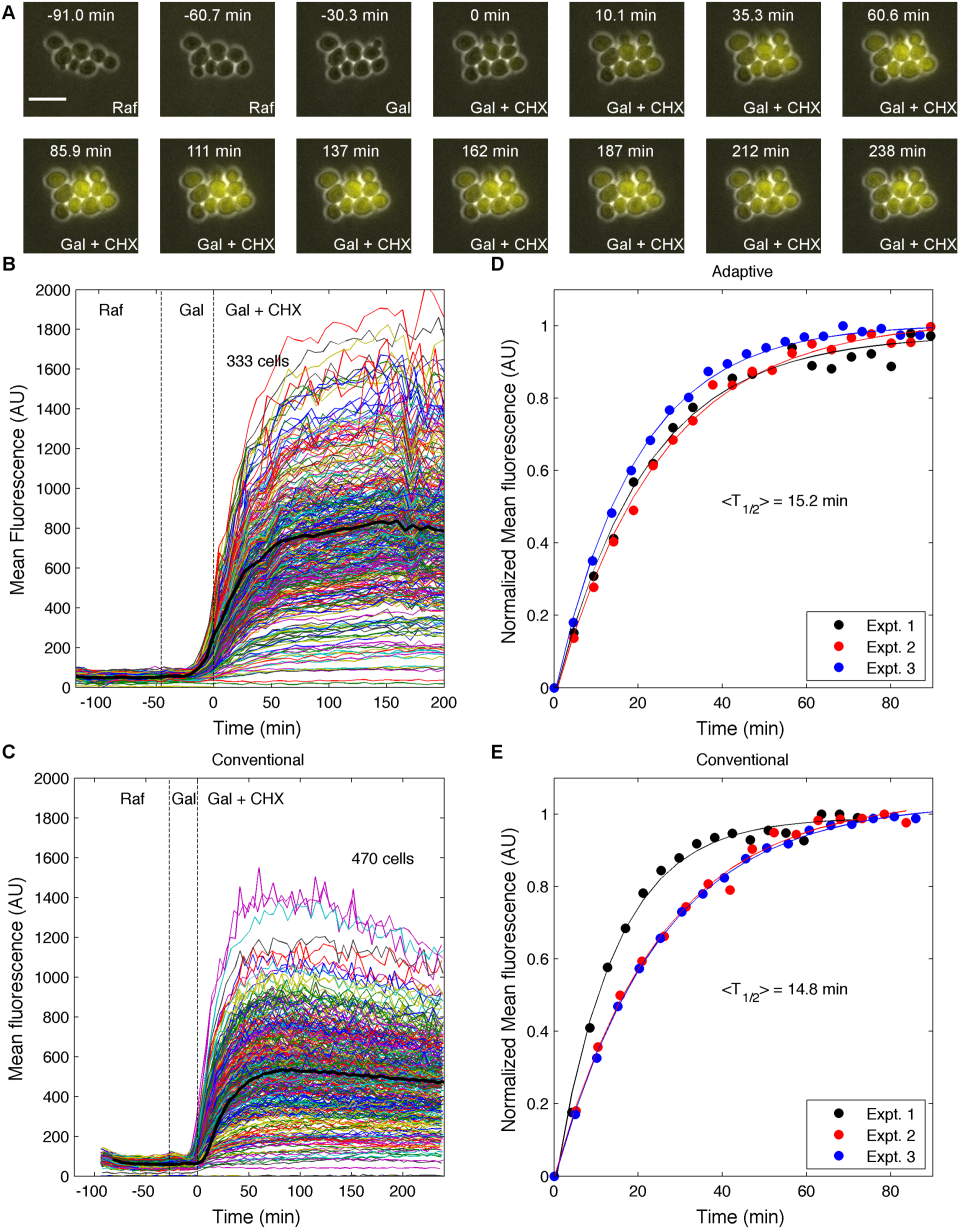
Characterizing the maturation of Venus. (A) Time-lapse images of a Venus maturation rate experiment showing budding yeast expressing Venus fluorescent protein under the control of the *GAL1* promoter. The cells were exposed to the indicated media: Raf, SC media+2% raffinose; Gal, SC media+2% galactose; Gal+CHX, SC media+2% galactose+20 µg/ml cycloheximide. Venus was induced by galactose addition at time −30.3 min and cycloheximide was added at 0 min to inhibit translation of new protein. Scale bar = 10 µm. (B and C) Time courses for individual cells subjected to galactose induction, and translation inhibition to characterize the maturation rate of Venus using (B) adaptive and (C) conventional fixed-time imaging. Each colored line represents data from a single cell, while the solid black lines show the average of the population. Dashed lines indicate the time at which media was changed to galactose, and cycloheximide (t = 0). (D and E) Fits of population averages to Equation 3 for (D) adaptive and (E) conventional fixed-time imaging. Experimental data shown as dots, and the corresponding colored line is the best fit to the maturation equation of that dataset.

Figure 4A shows a time course montage of images (taken from Movie S1) from one of the maturation experiments, with time 0 min being the time of addition of cycloheximide. Figure 4B and C show the time traces for individual cells in the adaptive and conventional imaging experiments, respectively.

For each experiment, the average trajectory of all cells (black lines in Figure 4B and C), *F*(*t*), was normalized to start at 0, and end at 1, and fit by least-squares to:
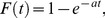
(4)where *a* is the maturation rate, which can be expressed as a half-time, *T_1/2_* = ln(2)/*a* [32].

When the population averages are fit to the model of FP maturation, the maturation rates from the adaptive experiments (*T_1/2_* = 15.2±1.1 min.) are similar to those from the conventional experiments (*T_1/2_* = 14.8±2.5 min., Table 1, Figure 4D and E). These values are well within the range of previous *in vitro* measurements (2 min. [37], and 40 min. [35]), and similar to previous measurements of Venus maturation performed in *S. cerevisiae* (11.2±1.6 min.) [33]. We also looked at an alternate analysis of this data by fitting each cell individually to Equation 4 (Table S1), which gave similar results to the single fits of the population averages reported in Table 1. Table 1 also presents the time at which the media changes occurred for each experiment (indicated by the vertical dashed lines in Figure 4B and C). The induction lasted roughly 1.5 times longer in the adaptive imaging mode, which results in a higher signal. Although this doesn’t result in a difference in maturation rates using Venus, this difference in induction time could affect measurements for a slower maturing fluorescent protein such as eGFP. Compared to the traditional approach, the time spent imaging after the addition of cycloheximide is roughly 60% as long in adaptive imaging mode and the overall movie was shorter by 50 minutes. Further, the conventional mode required an additional 4 hours of post-acquisition image processing, while this processing was complete at the end of the adaptive experiments.

It is interesting to note the variability in the timing of the cell growth and induction phases of the adaptive experiment. Although efforts were made to ensure that each experiment was initiated with cells in the same physiological state, the time required for the population to increase by 50% is quite variable, with a coefficient of variation (CV) of 20%, compared to the induction times (CV = 8%). The small variability in the timing of the phases in the conventional experiments (Table 1, columns 6 and 7) is due to differences in Δ*t_min_* between the experiments that result from the translation stage having to move differing distances to the various FOVs. One of the three conventional experiments resulted in an estimate of *T_1/2_*, that was ∼56% of the other two, which results in the higher standard error of the mean (SEM) as calculated across the three replicates. In contrast, the adaptive experiments returned quite consistent estimates across all three replicates.

The optimization of both the number of cells in each FOV, and the acquisition timing based on empirically measured hardware and software latencies to avoid microscope idle time has allowed us to consistently track >400 cells (Table 1) with a time resolution of 5 minutes even while analyzing the images as they are collected.

### Application 2: measuring transcription memory

In the above example, the adaptive control was used to change the growth medium based on the cells’ behaviors. We also performed more complicated experiments to demonstrate that it is possible to adaptively control both the growth medium and the imaging process. Specifically, it is possible to concentrate the limited imaging resources on the “interesting” members of the population. To demonstrate GenoSIGHT’s ability to change acquisition parameters along with environmental factors, and to investigate any artifacts introduced by changing the time-resolution during a time-course experiment, we have performed a series of 3 experiments using the *GAL1pr-Venus* strain. Starting with cells grown in repressing conditions (glucose), Venus was induced in galactose-rich media followed by an intervening period in which the population was exposed to glucose to repress *GAL1pr*-*Venus* transcription. After Venus levels dropped back down, Venus expression was re-induced by galactose. This experiment design was chosen due to the well-known increased induction rate that has been observed in cell populations during the second exposure to galactose, which has been dubbed transcriptional memory [39]–[42]. To the best of our knowledge, transcriptional memory has never been characterized at the single-cell level, most likely due to the difficulty in determining when media changes should occur. By using real-time image processing to automate the environmental changes and take the guess-work out the procedure, we have been able to demonstrate that transcriptional memory does indeed occur in individual cells, and not only at population level.

Like the maturation experiments, the cells were loaded into the microfluidic device, and the growth media was automatically changed based on the cells’ behavior. However, for this set of experiments, the cultures were grown overnight in SC+2% glucose to repress the *GAL1* promoter. After placing the microfluidic plate on the microscope, experiments consisted of 4 separate phases. Cells were first monitored in SC+2% glucose to ensure proper growth. The media was then changed to SC+2% galactose to induce expression of Venus until 50% of the cells showed ≥50% increase in their fluorescence. Then the media was switched back to SC+2% glucose to turn off Venus expression, and remained until the population doubled twice. Finally, SC+2% galactose was added to induce the expression of Venus again until 50% of the cells showed ≥50% increase in fluorescence, at which point the experiment was ended.

Each experiment was performed with a different acquisition strategy: 1) All FOVs were imaged during all four experimental phases with constant time resolution throughout; 2) Any FOVs that on their own did not have 50% of the cells increase by 50% during the first induction were dropped, and not imaged during the subsequent experimental phases, but the time resolution remained constant; 3) FOVs with <50% of cells showing an increase of 50% in fluorescence during the first induction were excluded from the subsequent phases (as in 2), and the time-resolution was adjusted to again maximize the amount of data collected (i.e., 8 of 30 FOVs were excluded, and so the time resolution was reduced from 4.6 to 3.4 minutes).

**Figure 5:**
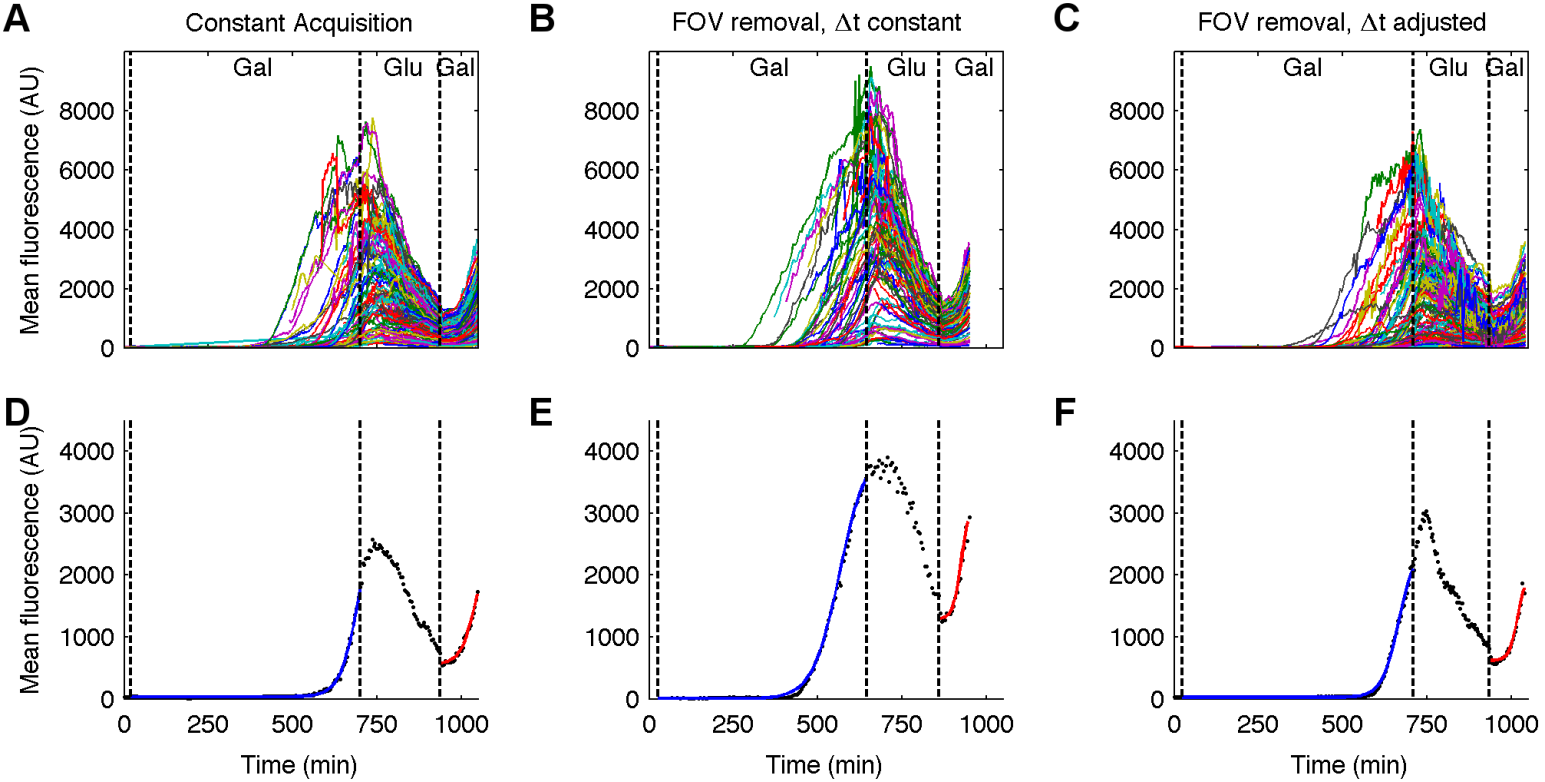
Transcriptional memory in single cells. (A–C) Single cell trajectories for experiments with (A) all FOVs imaged throughout the experiment with fixed timing intervals, (B) removal of FOVs containing less than 50% of cells exhibiting a fluorescence increase of ≥50%, with constant timing intervals, and (C) removal of FOVs as in (B), but with the timing intervals reduced from 4.6 minutes to 3.4 minutes during the second galactose induction, and the intervening period of glucose suppression. (D–F) Example fits to Equation 4 for single cells that were observed during both inductions. Black dots are the observed data, blue lines are the fits to the first induction, and red lines are the fits to the second induction.

Single-cell fluorescence trajectories for the three different experiments are shown in Figure 5A–C (see Movie S2 for an example of the time-lapse of 1 FOV). To quantify the sigmoidal shape of the inductions, the mean fluorescence for each cell, *F*(*t*), was fit with least-squares to the logistic function:
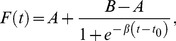
(5)


where *A* and *B* are the lower and upper asymptotes, respectively, *β* is the maximum rate at which fluorescence increases, and *t*_0_ is the time at which maximal increase occurs (i.e. when *F*(*t*) = (*B*+*A*)/2). We chose the logistic function over others, such as the Hill function, that can reproduce a sigmoidal shape due to the ease of interpretation of the various parameters for comparison between the two inductions. Figure 5D–F show fitting examples of cells that were detected during both inductions for each experiment, while the summary of all fitting is shown in Table 2. As expected due to transcriptional memory, all 3 experiments show that after the cells were exposed temporarily to galactose, and then returned to a glucose-rich environment, they remained in a prepared state, and responded more quickly to the second introduction of galactose. The time delay from the introduction of galactose to Venus production, *t*_0_, decreases by a factor of ∼6 during the reindunction, while the rate of fluorescence increase, *β* improves by a factor of ∼2 (Table 2). Although the work done by other groups did not attempt to quantify the rates of increase, our data does compare favorably well qualitatively with previous results, such as Figure 1C in [42] Due to the overall length of these experiments (∼17 hours), it was required to begin each with an extremely low cell density as reflected in the small numbers in Table 2.

**Table 2:** Summary of transcriptional memory experiments.

	First Induction				Second Induction			
	Δ*t*	Cells	Delay (*t*_0_, min) [SEM*]	Rate (*β*, min^−1^) [SEM*]	Δ*t*	Cells	Delay (*t*_0_, min) [SEM*]	Rate (*β*, min^−1^) [SEM*]
Constant Acquisition	4.0	22	553 [11.1]	0.04 [0.004]	4.0	175	98.8 [1.07]	0.06 [0.001]
FOV removal Δt = const.	5.0	28	559 [10.1]	0.03 [0.002]	5.0	73	77.1 [1.57]	0.08 [0.002]
FOV removal Δt adjusted	4.6	40	613 [11.5]	0.03 [0.002]	3.4	218	88.6 [0.67]	0.07 [0.001]

*SEM = standard error of the mean (N = number of cells).

## Discussion

Conventional imaging experiments consist of a highly linear workflow that involves manually selecting FOVs, setting fixed image acquisition parameters, image acquisition at predetermined time points, image processing and data reduction, and data analysis. By integrating the image analysis in the control loop, it is possible to adapt the image acquisition process at run time based on the behavior of the cells under observations. We have shown that this intelligent imaging strategy increases the quality of the data extracted from an experiment while reducing the time it takes to perform the experiment.

The process of manually identifying suitable FOVs is time consuming and can easily take 30 min of operator time even when using a small (3×3 mm) microfluidic chamber. There is no guarantee that the FOVs selected by the operator are optimal either. Automating this step frees the operator from this labor intensive and tedious task while ensuring the FOVs selected by the system meet some user-defined specifications. Considering that the ONIX system used in GenoSIGHT includes four chambers, saving 30 min of labor per chamber saves two hours of the operator workday, representing a gain of productivity of 25%. The FOV selection step could be based on other metrics than the one used here. For example, when doing transient transfections in mammalian cell lines, it is common to have a GFP to mark the cells that are transfected (usually only around 30% of the population). In this case, the operator would want to select FOVs based on fluorescent cells instead of cell numbers.

By moving the image processing and data analysis into the control loop with the acquisition, the operator can know immediately if an experiment is progressing as expected. It is often not possible to detect if cells are growing normally by just visually inspecting them. GenoSIGHT is capable of detecting that cells are not behaving as expected and notify the operator in real-time so that the experiment can be restarted immediately. In our laboratory, out of the last 30 experiments that were run adaptively, GenoSIGHT terminated 10 because the cells weren’t growing or did not express fluorescent protein as expected. Being able to detect failure early represents a 33% increase of productivity.

Another time saving benefit of adaptive control is the possibility of detecting the successful completion of an experiment. In many cases, operators will collect time-series longer than is necessary to support the goal of the experiment. Performing the data analysis at the source during the data analysis process increases the experiment throughput. The process of moving data from one computer to another, doing the image processing and data analysis was time consuming and error-prone. We estimate that the post-processing of images was taking about as much time as performing the experiments themselves. By handling this aspect of the workflow in real-time, we estimate that we have increased our productivity by 50%.

We estimate that GenoSIGHT has increased our productivity ten fold compared to what we could achieve using a state of the art commercial system relying on an open loop control of the imaging process. Because we can detect early if an experiment is not behaving as expected, we can reliably perform four experiments per workday. These experiments now take a single day instead of two days when the data analysis was performed in a post-processing phase. So, our throughput has increased from 2.66 successful experiments (assuming a 30% failure rate) to 8 experiments in two days. This corresponds to a 3-fold increase in throughput. Furthermore, the labor involved in performing these experiments has been reduced substantially now that the workflow has been completely automated. Loading the microscope and collecting the data of 8 experiments does not take more than 2 to 3 hours. When the data analysis was performed offline, it would take the best part of a work day and loading the microscope and finding the FOVs would still take 2 hours for 4 experiments. We can now perform three times more experiments with three times less effort (3 hours instead of 10 hours). Combining these two factors results in a 10-fold increase of productivity.

In addition to saving time and increasing productivity, adaptive control of the imaging process leads to more informative data sets than is possible using conventional instruments. The automated selection of FOVs allows the system to select the most usable FOVs therefore maximizing the number of cells observed while limiting the risks of collecting images that cannot be properly segmented. By adapting the changes of medium to the physiological state of the cells, it is possible to collect data that reduce the variability of parameter estimates by a factor 2 (Table 1). Finally, adaptive control allows operators to perform experiments producing data well adapted to estimate parameters of gene expression (Figure 5). Such experiment would be practically impossible to perform using conventional imaging systems.

Here, we have demonstrated the capabilities of GenoSIGHT in two types of gene induction experiments in yeast. We have also performed a few experiments in *E. coli*. Preliminary data show that algorithms must be tailored for the shape and size of the cells under observation, and this will affect the image processing latencies. GenoSIGHT modular architecture will make it possible to plug different image processing algorithms [43] suitable to track mammalian cells.

Our current implementation of adaptive imaging based on wide-field microscopy suffers a drawback in that any changes that are made in the acquisition affect entire FOVs rather than single cells. It would be preferable to be able to focus on individual cells exhibiting a particular behavior. However, there is currently no commercially available microscope system capable of performing this type of single cell acquisition, and would require the development of custom imaging hardware in order to collect data on each cell at different rates.

GenoSIGHT extensible architecture allows users to define their own functions to analyze cell properties like fluorescence, growth, shape, or intracellular distribution of proteins to any criteria defined by the user. For example, when monitoring the abundance of periodically expressed proteins, the system could fit the single-cell trajectories to a sine-wave, and use the extracted waveform frequency to adaptively adjust the experiment’s time resolution in order to minimize the cells’ light exposure while maintaining enough sampling points with which to fit the data. The experiment could be automatically stopped when the fit parameters have converged.

Adaptive imaging is model-driven. Since the data collection relies on an abstract description of the expected behavior of a cell population in specific experimental conditions, it is likely that the resulting data sets will be more suitable to precisely characterize the dynamics of gene expression but this requires the development of new statistical methods to properly analyze the data generated by this new generation of imaging systems.

## Supporting Information

Figure S1 **Comparison of image segmentation with CellTracer.** (A) The speed at which the image processing algorithm described in Materials and Methods (•) and the open source software, CellTracer (+) can identify all cells in an image containing the indicated number of cells. (B) The relationship between the actual number of cells in an image and the number automatically identified for the in-house software (•) and CellTracer (+). Red line indicates a slope of 1.
(TIF)

Table S1 **Statistics of maturation half-times extracted from single-cell trajectories.**
(DOCX)

Movie S1 **Example time-lapse movie of Venus maturation in yeast from a conventional, fixed time experiment.** Scale bar = 10 µm. Text indicates the time in minutes in relation to the addition of cycloheximide, and the growth media: Raf = SC+2% raffinose; Gal = SC+2% galactose; Gal+CHX = SC+2% galactose+20 µg/ml cycloheximide.
(MOV)

Movie S2 **Example time-lapse movie of transcriptional memory in the** ***GAL1*** **promoter in response to galactose using the protocol where FOVs were removed, and the time resolution was adjusted.** Scale bar = 10 µm. Text labels indicate the time in minutes from the beginning of imaging and the growth media: Glu = SC+2% glucose; Gal = SC+2% galactose.
(MOV)
