# Supplementary figures and images for: Adaptive Imaging Cytometry to Estimate Parameters of Gene Networks Models in Systems and Synthetic Biology
Source: PLoS One. 2014 Sep 11;9(9):e107087. doi: 10.1371/journal.pone.0107087 (PMC4161401; doi:10.1371/journal.pone.0107087)

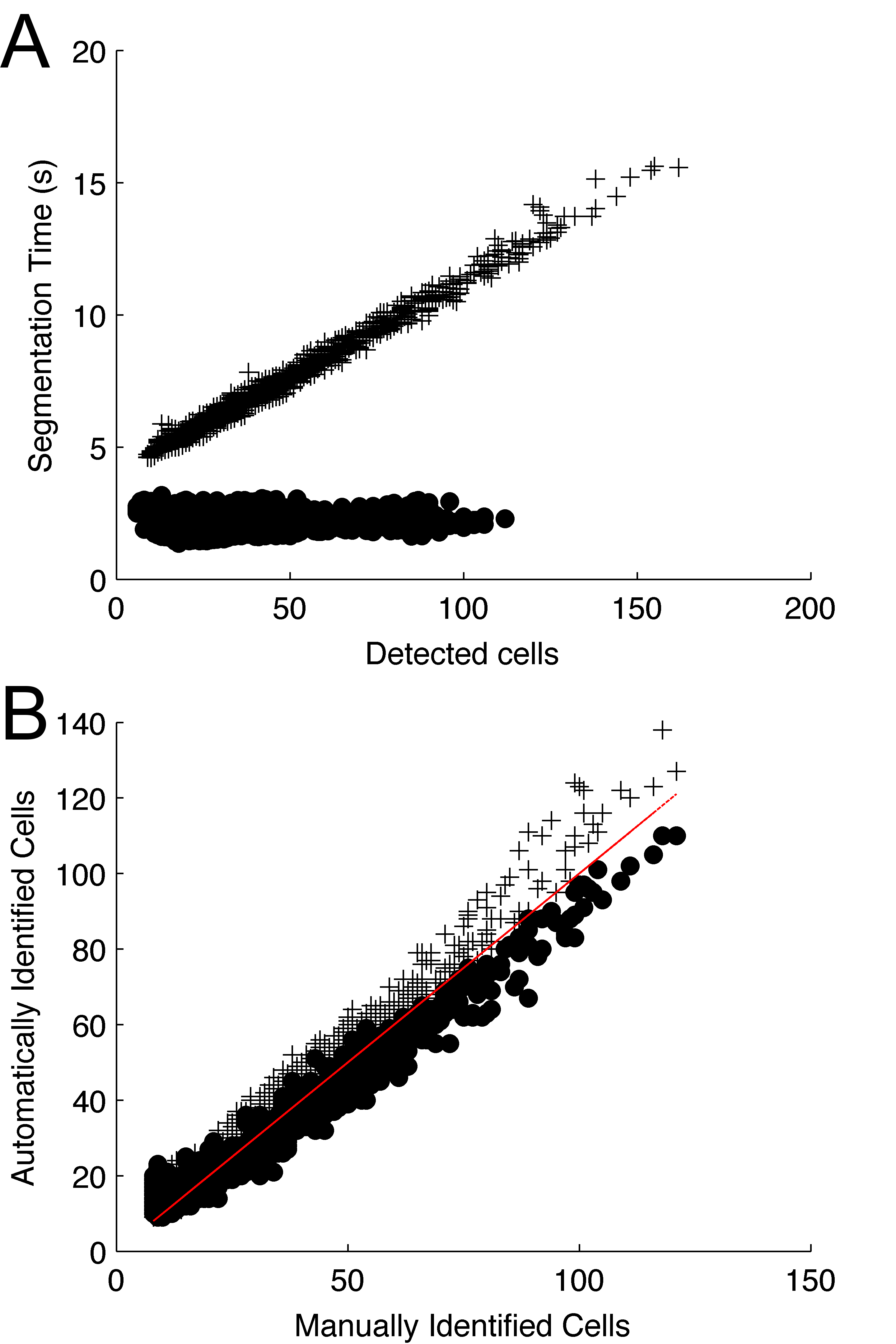

Supplement: Figure S1 — Comparison of image segmentation with CellTracer. (A) The speed at which the image processing algorithm described in Materials and Methods (•) and the open source software, CellTracer (+) can identify all cells in an image containing the indicated number of cells. (B) The relationship between the actual number of cells in an image and the number automatically identified for the in-house software (•) and CellTracer (+). Red line indicates a slope of 1. (TIF) [file pone.0107087.s001.tif]
