# Supplementary material for: Adaptive Imaging Cytometry to Estimate Parameters of Gene Networks Models in Systems and Synthetic Biology
Source: PLoS One. 2014 Sep 11;9(9):e107087. doi: 10.1371/journal.pone.0107087 (PMC4161401; doi:10.1371/journal.pone.0107087)
Supplement: Table S1 — Statistics of maturation half-times extracted from single-cell trajectories. (DOCX) [file pone.0107087.s002.docx]

Table S1: Statistics of maturation half-times extracted from single-cell trajectories

|  | Adaptive | | | Conventional Fixed Times | | |
| --- | --- | --- | --- | --- | --- | --- |
|  | Mean (min) | Standard Deviation (min) | N* | Mean (min) | Standard Deviation (min) | N* |
| Exp. 1 | 15.0 | 2.86 | 296 | 10.0 | 3.02 | 275 |
| Exp. 2 | 23.8 | 3.76 | 649 | 18.1 | 2.92 | 423 |
| Exp. 3 | 13.5 | 1.80 | 160 | 17.9 | 3.60 | 445 |

*number of cells after removing fits that never converge
